# Sleep, testosterone and cortisol balance, and ageing men

**DOI:** 10.1007/s11154-022-09755-4

**Published:** 2022-09-24

**Authors:** Peter Y. Liu, Radha T. Reddy

**Affiliations:** 1 Division of Endocrinology, Metabolism and Nutrition, Department of Medicine, Harbor UCLA Medical Center and The Lundquist Institute, 1124 W Carson St., Box 446, Torrance, CA 90502 USA; 2 David Geffen School of Medicine at UCLA, Los Angeles, CA USA

**Keywords:** Androgen, Glucocorticoid, Cardiometabolic health, Circadian, Sleep disordered breathing

## Abstract

Sleep serves important biological functions, and influences health and longevity through endocrine and metabolic related systems. Sleep debt, circadian misalignment and sleep disruption from obstructive sleep apnea is widespread in modern society and accumulates with life because recovery sleep is not completely restorative. Accumulated disordered sleep throughout life impacts the ageing process and the development of age-related diseases. When epidemiological and interventional studies are considered collectively, sleep loss and lower sleep duration are associated with lower morning, afternoon and 24-h testosterone; as well as higher afternoon, but not morning or 24-h cortisol. These reciprocal changes imbalances anabolic-catabolic signaling because testosterone and cortisol are respectively the main anabolic and catabolic signals in man. Fixing testosterone-cortisol balance by means of a novel dual-hormone clamp mitigates the induction of insulin resistance by sleep restriction and provided the first proof-of-concept that the metabolic harm from sleep loss can be ameliorated by approaches that do not require sleeping more. Obstructive sleep apnea is associated with lower testosterone, even after controlling for age and obesity whereas the conclusion that continuous positive airway pressure therapy has no effect on testosterone is premature because available studies are underpowered and better-quality studies suggest otherwise. High dose testosterone therapy induces OSA, but more physiological dosing may not; and this effect may be transient or may dissipate with longer term therapy. Studies investigating the origin of the diurnal testosterone rhythm, the effect of circadian misalignment on testosterone-cortisol balance, and methods to mitigate metabolic harm, are required.

## Introduction

Sleep restores neurobehavioral performance [1], improves immune function [2], and conserves whole body energy expenditure through metabolic processes that also restore brain energy stores and are important for neuronal plasticity and connectivity [3]. These sleep-related benefits on metabolism, immunity and cognition are essential for healthy ageing. Conversely, the accumulation of sleep debt across the lifespan negatively impacts metabolic conservation, neurobehavioral performance, immunity and autoimmunity – impacts that may be responsible for the development of diseases that accrue with age.

Sleep debt accumulates throughout life from repetitive episodes of insufficient sleep (not sleeping for an adequate amount of time), misaligned sleep (as occurs with jetlag or night shiftwork), and disrupted sleep (from obstructive sleep apnea, nocturia associated with ageing or sporadic environmental noise) [4]. This accumulation occurs because intermittent recovery or “catch-up” sleep does not seem to completely reverse the adverse effects of sleep debt on multiple physiological processes including psychomotor performance [1, 5], metabolism [6, 7], blood pressure regulation [8] and immune/adrenal response [9]. Furthermore, the accumulation of sleep debt is widespread in modern society: approximately one in three sleep insufficiently (< 7 h per night) [10], up to 20% are shiftworkers with work schedules that misalign sleep [11], and 10% of men (3% of women) aged 30–49 years and 17% of men (9% of women) aged 50–70 years have at least moderate obstructive sleep apnea [12]. Insufficient, misaligned and disrupted sleep is therefore likely to have major repercussions on ageing and the development of age-related diseases because catch-up sleep is ineffectual and abnormal sleep is widespread in our modern society. An important consideration in determining accumulated sleep debt is that sleep need itself decreases with older age. Another consideration is that the COVID-19 pandemic increased the global prevalence of sleep disturbances particularly in younger individuals infected with the disease, but whether this trend will persist is not yet known [13].

Sleep may influence health and longevity through endocrine and metabolic systems [14]. This is because endocrine networks evolved to regulate whole-body metabolism, including catabolism and anabolism, in a diurnally appropriate manner, and to simultaneously allow dynamic responses to external environmental insults and internal stress through the ultradian (also known as pulsatile) nature of hormone secretion [15, 16]. An important concept here is that the circadian pattern in hormones allows for anticipation of regular events that occur in a 24 h day (for example to regulate whole-body metabolism), whereas the pulsatile nature of hormone release results in large changes in hormone concentrations in a short period of time which allows for a more rapid and biologically-efficient (as opposed to a sustained non-pulsatile release) response to unpredictable events. In this fashion, specific endocrine networks can coordinate growth, puberty and reproduction in an age- and environment- appropriate manner.

**Fig. 1:**
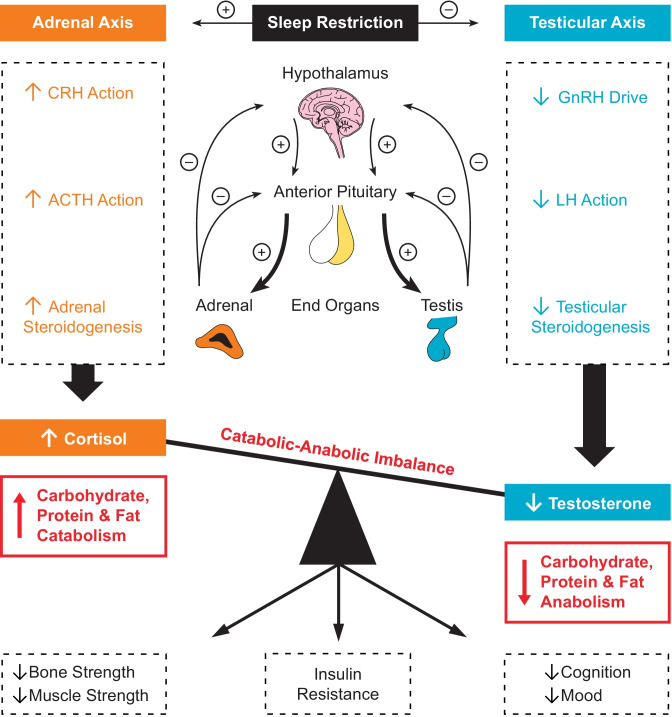
Sleep restriction imbalances cortisol and testosterone and induces insulin resistance. ACTH Adrenocorticotropic Hormone; CRH Corticotropin Releasing Hormone; GnRH Gonadotropin Releasing Hormone; LH Luteinizing Hormone

This review will primarily examine the effect of sleep disturbances on testosterone in the context of male gonadal ageing. However, to understand the full impact of restricted sleep on testosterone, it is necessary to determine the net effects on whole body metabolism and metabolic illnesses in conjunction with cortisol: see Fig. 1. This is because the hypothalamo-pituitary testicular and hypothalamo-pituitary adrenal axes that respectively control testosterone, the major anabolic hormone in men (but not women) and cortisol, a key catabolic signal, are intertwined in their regulation and sleep restriction imbalances both their signaling [4]. Obstructive sleep apnea (OSA) and its interaction with testosterone is of particular clinical relevance because sleep architecture is disrupted and sleep duration is reduced. The review will therefore conclude with a discussion of OSA and testosterone in the context of ageing.

## Sleep, sleep architecture, and ageing

Sleep is highly organized and is divided into periods of non-rapid eye movement (NREM) sleep and periods of rapid eye movement (REM) sleep. NREM sleep is subdivided into 3 stages (1, 2 and 3) and transitions from stage 1 to 3 represent progressive slowing of the electroencephalogram that occurs as sleep deepens from wake. Stage 3 NREM sleep, also named slow wave sleep (SWS), mostly occurs during the first half of the biological night and is the stage of sleep that is most metabolically and hormonally active [4]. On the other hand, REM sleep mainly occurs during the second half of the biological night and is required for memory consolidation and vivid dreaming. Sleep loss or disrupted sleep occurring at different biological times will therefore differentially affect sleep architecture. Since these different sleep stages likely serve different biological functions, the impact of sleep loss or disruption at different times of the biological night will also vary.

Expert opinion suggests that adults aged between 18–64 require 7–9 h of sleep per night, whereas adults over 65 need 7–8 h, including naps [17]. This is because sleep need decreases with age, but only up to 60 years, after which sleep need remains stable. Meta-analyses of cross-sectional studies of healthy adults show that total sleep time decreases by 10–12 min for each decade of life from age 20 to 60 years, but does not change thereafter [18]. Sleep architecture changes analogously over this age period: the proportion of SWS and REM sleep decreases while the proportion of stage 1 and 2 sleep increases before age 60, but is stable thereafter. An important limitation of the studies examining sleep architecture is the cross-sectional nature of the cohorts that were analyzed. Available longitudinal studies are inconclusive due to small sample size and short follow up. For example, one such study of 11 community dwelling seniors (8 women and 3 men) aged 60 to 72 years showed no changes in sleep architecture 3 years later, other than a small increase in the number of sleep stage transitions [19]. Large scale longitudinal studies are required to fully understand the changes in sleep architecture that occur with ageing, but are difficult to perform because in-laboratory polysomnography in large numbers of adults followed for decades is likely required to detect relevant changes.

## The inter-relationships between sleep duration, testosterone and ageing

### Testosterone and ageing

Many longitudinal and cross-sectional epidemiological studies of multiple populations across the world have unequivocally shown that testosterone declines with age in men [20]. More recently, the relevance of these declines in testosterone has been illustrated in the United Kingdom biobank cohort of 150,000 men followed for 11 years. Here, lower circulating testosterone concentrations were associated with higher future all-cause and cancer-related mortality [21]. Many of the features of ageing such as increased fat, insulin resistance, falls and fractures, and decreased muscle mass, muscle strength, physical performance, bone mineral density, erectile function and libido are also reminiscent of androgen deficiency when considered as a whole. Therefore the decline in testosterone, irrespective of the underlying reasons for the decline, ultimately leads to testosterone levels that are so low that it triggers consideration of the diagnosis of hypogonadism because suggestive clinical features, which overlap with non-specific features of ageing, are likely to be present already [22]. A syndrome of late-onset hypogonadism has since been objectively defined as requiring three sexual symptoms (decreased sexual interest, fewer morning erections and erectile dysfunction) in conjunction with a total testosterone below 11 nmol/liter and free testosterone below 220 pmol/liter [23] and is now widely accepted to properly identify individuals who may require testosterone replacement therapy [24]. However, this decline of testosterone with age is now recognized to be largely, although not exclusively, due to factors associated with ageing such as concomitant obesity and illnesses, rather than from ageing itself [20].

Notwithstanding these epidemiological conclusions, animal experiments and clinical investigations in healthy men have unveiled multiple alterations in the ageing hypothalamic-pituitary–testicular axis [25–27]. These regulatory changes include: (1) smaller amplitude Luteinizing Hormone (LH) pulses which suggest reduced drive by Gonadotropin-Releasing Hormone (GnRH) and/or excessive sex-steroid inhibition; (2) more frequent LH pulses and less orderly patterns of LH release, pointing to reduced negative feedback; (3) preserved LH response to exogenous GnRH pulses, revealing intact gonadotrope responsiveness; (4) reduced pulsatile and total daily testosterone secretion; and, (5) impaired testosterone secretion in response to both elevated endogenous LH concentrations (stimulated by flutamide, tamoxifen, GnRH or anastrozole) and infused pulses of recombinant human LH [28].

These regulatory alterations suggest that the decline in testosterone in older men reflects multisite failure of the GnRH-LH-testosterone axis. Dissecting the individual signaling failures that together result in this set of findings, requires an integrative biomathematical model of reciprocal dynamic interactions among GnRH, LH and testosterone via estimable feedback and feedforward interactions [29]. This ensemble model reconstructs ageing-related adaptations among all three interlinked signals simultaneously, rather than appraising any one signal in isolation [30]. According to this ensemble model, the most parsimonious explanation of available data in humans is that ageing (1) diminishes hypothalamic GnRH outflow, (2) impairs testicular responsiveness to LH pulses and (3) decreases androgenic negative feedback [30, 31].

Experimental verification of the findings from ensemble modeling was recently achieved in 40 community dwelling men aged 19 to 73 years with body mass indices from 20.0 to 34.3 kg/m^2^ in whom multiple mechanisms were explored in the same individual for the first time [32]. Each individual underwent sequential testing in random order of hypothalamic GnRH outflow by administering a submaximal dose of ganirelix (a GnRH antagonist), hypothalamopituitary feedback by steroidogenic blockade with ketoconazole, and testicular responsivity with repeated pulsatile infusions of recombinant human LH [32]. Advancing age was shown to be associated with (1) diminished GnRH outflow, and not decreased GnRH responsivity of the gonadotrope, (2) diminished testicular responsivity to infused LH pulses and (3) partial compensation by reduced central gonadotropic response to testosterone feedback. A possible unifying mechanism that could underpin these diverse associations is that specific proinflammatory cytokines could induce androgen depletion simultaneously through enhanced inhibition of GnRH and/or LH secretion as well as direct Leydig cell effects [33, 34]. In any case, longitudinal studies will be required to establish the magnitude, relative importance and timing of inferred regulatory deficits in healthy ageing men. This is because declines in testosterone per year of age is 2–threefold greater with longitudinal compared with cross-sectional analysis of the same cohort [35, 36]. However, longitudinal studies examining for any of these regulatory changes are not available and attempts to re-examine an adequate number of individuals after a sufficiently long period of time have not been successful.

### Sleep duration, testosterone, and aging

A consistent relationship between sleep duration and testosterone has not been apparent in large cross-sectional studies of healthy community-dwelling older [37, 38] and young and older [39] men when divided according to studies that objectively measured sleep by actigraphy (a method to infer sleep from wrist-worn devices that detect motion) or polysomnography [37] from those obtained by self-report [38, 39]. Longitudinal studies comparing changes in sleep duration with changes in testosterone would be valuable to resolve whether a relationship exists, but are not available in men of any age.

Table 1 shows all the in-laboratory studies of sleep restriction on blood testosterone, divided into the 3 studies that assessed testosterone for a full 24 h [40–42], from those that did not [43–51]. Within each division, studies are ordered by more frequent blood sampling and then by larger sample size. Only one study intensively sampled blood frequently enough to allow determination of pulse characteristics by mathematical deconvolution [40]. All studies when collectively examined show that sleep restriction decreases testosterone. An important limitation is that most of these studies assessed testosterone only in the morning. Another limitation is that only 3 studies have examined 24-h testosterone, and of these, one [42] was confounded by strenuous exercise, which is known to increase testosterone [52]. Accordingly, understanding time of day differences is also necessarily limited.

**Table 1:** Effect of In-Laboratory Sleep Restriction on Morning, Afternoon and 24-h Testosterone

**Study**	**Subjects** **(n)**	**Age (yrs.)** **Mean ± SD;** **BMI (Kg/m**^**2**^**)** **Mean ± SD**	**Study Design**	**SLEEP OPPORTUNITY**				**Time of Testosterone Measurement**	**Results of Testosterone Levels**		
				**CONTROL** **# Of Days x Hours/day**		**SLEEP RESTRICTION** **# Of Days x Hours/day**					
				**Days**	**Hours**	**Days**	**Hours**		**AM**	**PM**	**24 Hours**
**Studies that Assessed Testosterone for 24 Hours**											
Liu et al. [40]	17 M (“young men”)	24.1 ± 2.9; Median 25 (IQR 22.9–27.5)	Randomized crossover	1	8 2200–0600	1	0	Q10 min × 24 Hrs 1800–1800	⬇ 0600–0900	⬇ 1500–1800	⬇
	18 M (“older men”)	63.9 ± 4.0; Median 29.5 (IQR 26.4–31.7)	Randomized crossover	1	8 2200–0600	1	0	Q10 min × 24 Hrs 1800–1800	⬇ 0600–0900	⬇ 1500–1800	⬇
Leproult and Van Cauter [41]	10 M	24.3 ± 4.3; 23.5 ± 2.4	Fixed order	3	10 2200–0800	8	5 0030–0530	Q15-30 min × 24 Hrs 1400–1400	NR	⬇ 1400–2200	⬇ 0800–2200
Dattilo et al. [42]	10 M Undergoing strenuous exercise	24.5 ± 2.9; 22.7 ± 2.3	Randomized crossover	3	8 2300–0700	2	0	Q2 Hrs. × 24 Hrs 1900–1900	NR	NR	⬌
**Studies that Assessed Testosterone for less than 24 Hours**											
Schmid et al. [43]	15 M	27.1 ± 5.0; 22.9 ± 1.2	Crossover, Balanced order	2 (Allowed to leave lab 0700–2000)	8 2245–0700	2 (Allowed to leave lab 0700–2000)	4 0245–0700	0740, then Q1 Hrs 0800–2300	⬌	⬌	⬌
Schmid et al. [43]	8 M	24.5 ± 3.1;	Randomized balanced order, 3-period	1	7 2200–0600	1	0	0700	⬇	NR	NR
		23.7 ± 1.7		1	7 2200–0600	1	4.5 2200–0330	0700	⬇	NR	NR
Reynolds et al. [44]	14 M	27.4 ± 3.8; 23.5 ± 2.9	Fixed order	2	10 2200–0800	5	4 0400–0800	0900, then Q2 Hrs 1000–2000	⬌	⬌	NR
Lamon et al. [45]	7 M	22 ± 1.8; 22.6 ± 4.1	Randomized crossover	1 (At home)	9 2200–0700	1	0	0700, 1000, 1300, 1500	NR	NR	⬇
	6 F	20 ± 1.3; 20.7 ± 3.2							NR	NR	⬌
Amal et al. [46]	14 M	31.4 ± 3.9; 24.0 ± 2.0	Randomized fixed order, crossover	6 (Last night in-lab)	8–8.5 2330–0700 “Habitual sleep”	1	0	0700, 1700	⬇	⬌	NR
				6 (Last night in-lab)	10 2100–0700 “Habitual sleep”	1	0	0700, 1700	⬇	⬇	NR
Sauvet et al. [47]	16 M	27.3 ± 5.4; 23.6 ± 0.6	Fixed order	1	8 2300–0700	1	0	0700	⬇	NR	NR
Carter et al. [48]	14 M	22 ± 1; 25.5 ± NR	Randomized order	1(at home) (Home actigraphy)	7.3 ± 0.2 On preceding nights Usual sleep	1	0	0730	⬇	NR	NR
	13F	22 ± 1; 22.8 ± NR							⬌	NR	NR
Akerstedt et al. [49]	12 M	NR (Range 19–30); NR	Fixed order	1	NR “Habituation night”	2	0	0800	⬇	NR	NR
Smith et al. [50]	11 M	36.6 ± 5.6; 24.2 ± 1.1	Randomized crossover	5	9 2000–0700	3–5	4 0100–0500	0730	⬌	NR	NR
Jauch-Chara et al. [51]	10 M	25.3 ± 4.4; NR (Range 20.7–25)	Balanced order	1	~ 7 1030–0530	1	0	0700	⬇	NR	NR

The study subjects are healthy. Studies are in-laboratory unless otherwise stated. All testosterone measurements are of total testosterone from serum unless otherwise stated

*AUC* area under the curve, *BMI* body mass index, *Hrs*. hours, *F* females, *IQR* interquartile range, *Kg* kilogram, *m* meter, *M* males, *Min* minutes, *NR* not reported, *Q* every, *SD* standard deviation, *T* testosterone, *yrs.* years

^#^ - number, ⬌ - No Change, ⬇ - Decreased, ⬆ - Increased

Only one study has examined the effect of manipulating sleep in a cohort that specifically included older men [40]. In this study, 18 healthy older men (average age 63.9 ± 4.0) as well as 17 healthy young male adults (average age 24 ± 2.9 years) underwent total sleep deprivation (complete nighttime wakefulness) and 8 h of regular night sleep in random order. Blood was sampled at a sufficiently high frequency, every 10 min, to allow unbiased, accurate and validated calculations by mathematical deconvolution of the timing and frequency of pulses; the mass per pulse; the basal, total and pulsatile secretion; and its biexponential elimination [15, 53–55]. These pulse characteristics contain information that is crucial for signaling between endocrine glands [15, 16, 53]. Blood was also sampled over an entire 24 hour period to allow appraisal of time of day and diurnal effects.

Whereas 24 h, morning, and afternoon testosterone concentrations were all decreased in both older and younger men by sleep restriction, a significant age group by sleep condition interaction revealed that sleep restriction decreased the testosterone pulse frequency and pulsatile secretion only in older men [40]. Twenty four-hour LH concentrations and pulse characteristics did not change despite these changes in testosterone. However, morning but not afternoon, LH concentration, pulsatile secretion and mass per pulse were reduced by sleep restriction in both young and older men.

## The inter-relationships between sleep duration, cortisol and ageing

### Cortisol and ageing

The 24-h rhythm of cortisol expressed in humans is driven by the central circadian pacemaker located in the suprachiasmatic nucleus [56, 57], which entrains all other peripheral clocks throughout the body to coordinate bodily processes with the environment [58, 59]. This rhythm is important for its action because cortisol, not melatonin, is recognized to be the key *metabolic* central synchronizing signal [60–62]. Melatonin is not discussed further in this review because cortisol, not melatonin, is the signal that has been shown to synchronize the timing of peripheral clocks in organs relevant for metabolism to the timing of the central circadian pacemaker. Experimental administration of glucocorticoid synchronizes the peripheral clocks located in liver, muscle, and adipose tissue which are the major storage sites for glycogen, protein, and fat, respectively, as well as other metabolically relevant organs such as pancreas and gut [60–63]. Resetting of the peripheral clock occurs through well-defined molecular mechanisms that involve direct interaction of glucocorticoids, glucocorticoid response elements and the regulatory regions of core clock genes *Bmal1, Cry1, Per1* and *Per2* [63, 64]. Therefore, understanding the 24-h pattern of cortisol, and not just averaged levels, is required. Documenting these changes requires high fidelity sampling of circulating cortisol patterns across an entire 24-h day in large cohorts with expansive age ranges. Only two such studies, of 143 and 177 healthy men and women aged 20 to 80 years, respectively, are available [65, 66].

These [65, 66] and other [67] studies are consistent in showing that age is associated with higher late-afternoon and early-evening cortisol, and an advance in the timing (*i.e.* phase) of the cortisol rhythm, but not with cortisol-binding protein [68]. These findings are highly relevant because hypercortisolemia during the late-afternoon and early-evening is harmful, and is believed to cause the insulin resistance observed with advanced age [60, 69, 70], impair physical performance [71] and trigger neurocognitive deficits [72]. In fact, experimentally increasing late-afternoon and early-evening cortisol through hydrocortisone administration induces insulin resistance the following day [69, 73, 74]. Premenopausal women, but not older women, had lower 24-h, and morning, cortisol concentrations compared with age-matched men [65, 66]. In contrast to the consistent findings previously outlined, 24-h cortisol concentrations were independent of age in one study [65], but increased with age in the other [66], for reasons that are not entirely apparent.

Only one of these two studies examined the effect of age on pulsatile cortisol secretory characteristics [65]. These analyses show that cortisol pulse frequency was higher, cortisol half-life was shorter, and basal secretion was lower in women compared to men, whereas total and pulsatile cortisol secretion did not differ by sex. None of these secretory parameters differed according to age in a multivariate analysis that adjusted for sex and obesity.

### Sleep duration, cortisol and ageing

Recently it has been established that actigraphically-measured sleep duration is not associated with higher daytime salivary cortisol levels (average of 3 samples collected from 9AM to 6PM) in a random subsample of 672 adults, representing a nationally representative probability sample of adults aged 62–90 years in the United States of America [75]. This is consistent with another study of 325 community-dwelling men aged 65 years or more that reported no significant association between actigraphally-measured sleep duration and 24-h urinary free cortisol [76]. These studies suggest that sleep duration does not influence 24-h or near 24-h cortisol. On the other hand, recurrent self-reported short sleep duration is associated with the development of higher late afternoon or bedtime salivary cortisol after 10 years of follow up in a large occupational cohort of 3314 adults initially aged 35–55 years [77]. These epidemiological data show that the effect of short sleep may be on a specific time of day (*i.e.* late-afternoon and early-evening) where higher cortisol levels result in metabolic harm, without any impact on 24-h cortisol. These findings are consistent with studies where sleep was manipulated: Table 2.

**Table 2:** Effect of In-Laboratory Sleep Restriction on Morning, Afternoon and 24-h Cortisol

**Study**	**Subjects** **(n)**	**Age (yrs.)** **Mean ± SD;** **BMI (Kg/m**^**2**^**)** **Mean ± SD**	**Study** **Design**	**SLEEP OPPORTUNITY**				**Cortisol Measurement Frequency and Duration**	**Results of Cortisol Levels**		
				**CONTROL** **# Of Days x Hours/day**		**SLEEP RESTRICTION** **# Of Days x Hours/day**					
				**Days**	**Hours**	**Days**	**Hours**		**AM**	**PM**	**24 HR**
Liu et al. [40]	17 M “Young men”	24.1 ± 2.9;	Randomized order	1	8 2200–0600	1	0	Q10 min × 24 Hrs 1800- 1800	⬌	⬌	⬌
		Median 25 (IQR 22.9–27.5)							0600–0900	1500–1800	
	18 M “Older men”	63.9 ± 4.0;	Randomized order	1	8 2200–0600	1	0	Q10 min × 24 Hrs 1800–1800	⬌	⬆	⬌
		Median 29.5 (IQR 26.4–31.7)							0600–0900	1500–1800	
Broussard et al. [78]	19 M	23.5 ± 3.1;	Randomized order	4	8.5 2300–0730	4	4.5 0100–0530	Q15-30 min × 24 Hrs 2130 – 2130	NR	⬆	NR
		23.4 ± 1.7								1900–2130 2300–0100	
Nedeltcheva et al. [79]	6 M 5 F	39 ± 5;	Randomized order	14	8.5 $\sim$2315- ~0740	14	5.5 ~ 0030- ~ 0600	Q15-30 min × 24 Hrs 2000 – 2000	⬌	⬆	⬌
	Sedentary	26.5 ± 1.5							AM peak Cortisol	2000–2200	
Leproult and Van Cauter [41]	10 M	24.3 ± 4.3; 23.5 ± 2.4	Fixed order	3	10 2200–0800	8	5 0030–0530	Q15-30 min × 24 Hrs 1400 – 1400	NR	NR	⬌
Vgontzas et al. [80]	12 M 13 F	M: 25.6 ± 4.1; 24.6 ± 1.5 F: 24.8 ± 3.4; 23.1 ± 2.7	Fixed order	4	8 2230–0630	8	6 2230–0430	Q30 min × 24 Hrs 0800 – 0730	⬇ AM peak Cortisol	⬌	⬌
Wright et al. [57]	14 M 3 F	31.7 ± 6.1; NR	Fixed order	6	8 (Habitual sleep)	1	0	Q30 min × 24 Hrs 2400 – 2400	⬆ $\sim$0900–1100	⬆ $\sim$1300, $\sim$1600	⬆
Pejovic et al. [81]	16 M 14 F	24.7 ± 3.5; 23.6 ± 2.4	Fixed order	4	8 2230–0630	6	6 2230–0430	Q1 Hr. × 24 Hrs 0800 –0800	⬇ AM peak cortisol	⬌	⬌
Ackermann et al. [82]	12 M	23 ± 5; NR	Fixed order	1	8 2300–0700	1	0	Q1Hr. × 24 Hrs 1200 – 1200	NR	NR	⬌
Benedict et al. [83]	14 M	22.6 ± 3.0; 23.9 ± 1.9	Randomized balanced order	1	8 2300–630-0700	1	0	1800, 2100, then Q90 min 2400 – 0900, Q60 min 1000 –1300, then 1500 & 1800	⬇ 0730 ⬆ 0900	⬆ 1200, 1500	⬆
Axelsson et al. [84]	9 M	Range 23–28; Range 21–26	Fixed order	2	8 2300–0700	5	4 0300–0700	Q1 Hrs 2300 – 0800, then Q3 Hrs 0800 – 2300	NR	⬆ 2000	⬌
Simpson et al. [9]	8 M 8 F	24.9 ± 4.4; 24.8 ± 3.2	Randomized balanced order	5	8 2300–0700	5	4 0300–0700	Q2 Hrs. × 24 Hrs 2330 – 2130	⬆ 0730	⬌	NR
Dattilo et al. [42]	10 M Undergoing strenuous exercise	24.5 ± 2.9; 22.7 ± 2.3	Randomized order	2	8 2300–0700	2	0	Q2 Hrs. × 24 Hrs 1900–1900	NR	NR	⬆

*BMI* body mass index, *F* females, *Hrs.* hours, *IQR* interquartile range, *Kg* kilogram, *m* meter, *M* males, *Min* minutes, *NR* not reported, *Q* every, *SD* standard deviation, *SR* sleep restriction, *Wk.* week, *Yrs.* years

^#^ - number, ⬌ - No Change, ⬇ - Decreased, ⬆ - Increased

Table 2 summarizes the twelve in-laboratory studies that have examined the effect of sleep restriction on blood cortisol determined over a full 24-h period, ordered by more frequent blood sampling and then by larger sample size [9, 40–42, 57, 78–84]. Twenty four-hour studies are essential because cortisol levels at specific times, particularly the late afternoon or early evening, are important for metabolic health. For this reason, studies of less than 24-h are not discussed.

When appraised collectively, these studies show that sleep restriction increases afternoon cortisol: Table 2. Whereas 6 studies reported increased afternoon cortisol with sleep restriction [40, 57, 78, 79, 83, 84], only 3 studies reported no change [9, 80, 81], and none reported a decrease. Furthermore, it has been directly shown that insufficient blood sampling of cortisol increases variability of averaged cortisol and reduces power [65] so that only studies of sufficiently high frequency of sampling may be adequately powered. Also, sleep loss may need to be of sufficient magnitude to induce cortisol changes. In this regard, *all* studies that sampled blood more frequently than every 30 min [40, 78, 79] or restricted sleep to 5.5 h/night or less [40, 57, 78, 79, 83, 84] reported that sleep restriction increased afternoon cortisol.

In contrast, sleep restriction does not seem to alter 24-h cortisol: Table 2. Seven studies reported no change in 24-h cortisol with sleep restriction [40, 41, 79–82, 84], 3 reported an increase [42, 57, 83] and none reported a decrease. The effect of sleep restriction on morning cortisol is not interpretable because different studies show increases, decreases or no change: Table 2. These discrepancies may be due to confounding by the cortisol awakening response which differs among studies according to the timing of scheduled wake and is of short duration so that confounding is compounded by infrequent sampling. With this in mind, all studies that sampled blood more frequently than every 30 min [40, 78, 79] actually show no change in morning cortisol.

Relevant for understanding the effects of ageing, only one study has included a cohort of older men [40]. This study is also the only study of sufficient sampling fidelity to allow calculation of pulsatile secretory parameters by mathematical deconvolution. Total sleep loss did not alter any pulse characteristic when assessed over the full 24-h period or in the morning (0600 to 0900), but analysis of a specific 3-h time window in the afternoon (1500 to 1800) revealed an increase in cortisol concentrations and pulsatile secretion specifically in older men. One limitation of this study is that the afternoon time window was relatively early. Older men have a phase-advanced acrophase of almost 3 h, so the 1500 to 1800 time period represents a later circadian time compared to young men [67]. This may explain why sleep restriction did not alter early afternoon cortisol in the young men examined in this study. Accordingly, if sampling had continued for an additional 3 h in the young men in this study (i.e. from 1800 to 2100), then increases in cortisol levels with sleep deprivation might have been detected, as it has been in other studies [85].

## Testosterone and cortisol imbalance from sleep restriction leads to metabolic harm

Cortisol and testosterone are respectively the major catabolic and anabolic signals in men [4]. Changes in cortisol and testosterone signaling from sleep restriction, namely a reduction in testosterone and an increase in late-afternoon and early-evening cortisol, has long been postulated to be a mechanism by which sleep restriction induces insulin resistance: see Fig. 1. Although the hypothalamo-pituitary testicular and hypothalamo-pituitary adrenal alterations that cause these changes in testosterone-cortisol balance are yet to be fully elucidated, substantial evidence implicate testosterone and cortisol in the development of insulin resistance. Randomized controlled trials show that testosterone treatment improves insulin sensitivity in men at risk for type 2 diabetes mellitus (T2DM) [86, 87]; prevents the development of T2DM in men at risk for T2DM [88]; and, improves glycemic control in men with T2DM [89]. Administration of glucocorticoids to bolster cortisol levels in the late afternoon and early evening also induces insulin resistance [69, 73, 74] through post-receptor mechanisms [90–92]. Nevertheless, direct substantiation of the role of testosterone-cortisol balance in the induction of insulin resistance from sleep restriction has only recently become available in a study that utilized a novel dual hormonal clamp [93].

Thirty-four healthy young mostly overweight men completed a randomized two-condition crossover study in a highly controlled in-laboratory environment [93]. Under both conditions, participants underwent one night of 10 h baseline sleep (2200–0800) followed by 4 consecutive nights where sleep was restricted to a 4 h opportunity (0100–0500). Under the dual hormonal clamp condition, mid-physiological cortisol and testosterone concentrations were maintained by co-administration of ketoconazole to block endogenous steroidogenesis in conjunction with simultaneous addback of physiologically appropriate doses of oral hydrocortisone and transdermal testosterone gel. In the placebo condition (where matching placebos for ketoconazole, hydrocortisone and testosterone were administered), cortisol and testosterone were not clamped. Fixing testosterone-cortisol balance mitigated the development of insulin resistance and hyperinsulinemia with 4 consecutive nights of sleep restriction by at least 50%, but did not prevent the development of hyperglycemia [93]. Future studies will be required to determine the relative contribution of testosterone and cortisol in inducing these metabolic changes, by individually clamping each hormone.

These findings provide first proof of principle for the amelioration of metabolic harm from sleep restriction by a targeted approach that does not require sleeping more. These findings are highly relevant to ageing because (1) this pattern of sleep restriction (4 nights of 4 h/night sleep opportunity) is ecologically valid, and (2) accumulated sleep debt over a lifetime could also lead to prediabetes and type 2 diabetes mellitus through the induction of insulin resistance; obesity due to hyperinsulinemia; and retinopathy and nephropathy due to hyperglycemia. Furthermore, fixing testosterone-cortisol balance in older men to prevent the development of insulin resistance and hyperinsulinemia is plausible because sleep restriction is now known to decrease testosterone and increase late-afternoon and early-evening cortisol in older men [40].

## Effect of circadian misalignment on testosterone and cortisol

Although the 24-h rhythm of cortisol is driven by the central circadian pacemaker [56, 57], the origin of the 24-h pattern of testosterone in humans is not known. Prior studies have documented a marked 24-h rhythm in testosterone [94–96], but these rhythms could be attributable to external rhythmic influences because cycles of light/dark, sleep/wake, feeding/fasting, and activity/rest were present. Showing that the 24-h pattern of testosterone is driven specifically by the central circadian pacemaker, free of external influences, requires the use of the constant routine protocol as the gold standard method to remove or uniformly distribute these external influences so that only the endogenous rhythm is expressed [97, 98]. These findings will have implications for the assessment of hypogonadism, including late onset hypogonadism, in night shiftworkers.

Circadian misalignment occurs when the timing of behavioral rhythms differs from the timing of endogenous circadian rhythm. It occurs with jetlag and repetitively in night shiftworkers who typically revert back to a day-aligned schedule (to interact with family and friends, address domestic duties, attend daytime events, etc.). Circadian misalignment is harmful. It induces insulin resistance, and night shiftwork is associated with metabolic diseases such as obesity and T2DM [4]. The effect of circadian misalignment on endocrine rhythms has not been adequately explored. Only one recent study utilized a constant routine to properly evaluate the effect of circadian misalignment on cortisol rhythms and concluded that 21 days of circadian misalignment through forced desynchrony reduced overall cortisol (24-h area under the curve) by 120 mcg/day [57]. No equivalent studies are available to examine the effect of circadian misalignment on testosterone rhythms. Such studies would be valuable because symptoms consistent with hypogonadism are common in night shiftworkers, particularly those with shiftwork sleep disorder [99–101], and understanding whether circadian misalignment per se can directly alter testosterone levels and induce hypogonadism would inform treatment strategies.

## Obstructive sleep apnea

### OSA, obesity and testosterone in ageing men

OSA is the most important clinical cause of disrupted sleep. It is a male-preponderant disease that is more prevalent with advanced age, particularly in the presence of obesity [12, 102]. Accordingly, low testosterone concentrations, OSA and obesity are known to frequently occur together, but the mechanisms that underpin these inter-relationships are not well established [103]. Cohort studies that include older men show that higher degrees of hypoxemia, as a marker of more severe OSA, are associated with lower testosterone concentrations [37, 104–106]. This association may [37] or may not [104–106] be due to obesity. A recent meta-analysis of 18 studies involving 1823 men with or without OSA also found a significant inverse relationship between OSA and testosterone concentrations – a relationship which remained after controlling for age and obesity [107]. Notably, a significantly lower testosterone was only observed in men with severe OSA. These findings collectively suggest that OSA, through hypoxemia or possibly through disrupted sleep architecture, most likely lowers testosterone, independently of obesity and age – although the reverse relationship is possible since these are cross-sectional analyses. The direction of this relationship would be better substantiated if reversing OSA with continuous positive airway pressure (CPAP) therapy increases testosterone.

However, the two available meta-analyses have not concluded that CPAP therapy increases testosterone in men with OSA, but have instead concluded that CPAP has no effect on testosterone [108, 109]. This conclusion is particularly problematic because these meta-analyses are likely underpowered, and meta-analyses consider all studies equally irrespective of quality. A notable limitation is that none of the available studies have assessed the effect of CPAP on 24-h testosterone. In fact, only one study assessed testosterone frequently, and actually reported that CPAP increased mean, incremental and area under the curve testosterone measured every 20 min during a 12-h period from 1900 to 0700, [110]. Although treatment duration was particularly long at 9 months compared with other studies, important limitations include self-selection to prolonged compliant CPAP therapy in a cohort that only included 5 men. The remaining 11 studies examined the effect of CPAP on morning testosterone, assessed on one or two occasions only, again in only relatively few men [104, 111–120]. Of these studies, only one is a randomized sham-controlled trial, and this study actually reported that CPAP improved testosterone compared with sham – although this was due to a reduction in testosterone in the sham group rather than an increase in testosterone in the treated group [112]. Not considered in any of these meta-analyses is a complementary study of 12 men in whom sleep disordered breathing was almost completely reversed for the entire duration of sleep through surgical uvulopalatopharyngoplasty [121]. Blood testosterone significantly increased after 3 months, while the number of apneas fell from an average of 40 events/hour to 5 events/hour.

Due to the limitations of the available literature, it seems premature to conclude that CPAP has no effect on circulating testosterone levels in men with OSA. The available meta-analyses included no more than 12 studies (of which 2 were randomized controlled trials), and the higher quality studies and other complementary data suggest that CPAP increases testosterone. Nevertheless, it is a plausible that CPAP has no effect on circulating testosterone levels, which if true would point to irreversible neuroendocrine changes to the testicular axis arising from longstanding intermittent hypoxia – irreversible changes that are not found in the adrenal axis. By comparison, a meta-analysis of 22 studies (of which 6 were randomized controlled trials) was sufficiently powered to conclude that CPAP decreases cortisol [122]. More studies, including randomized sham-controlled studies utilizing repetitive overnight or 24-h blood sampling and modern mathematical deconvolution techniques that better discriminate subtle regulatory changes are needed to finally resolve this issue.

### Testosterone effects on OSA

Testosterone therapy is widely believed to induce or worsen sleep apnea, and recent European and North American societal guidelines recommend vigilance in the detection of new OSA, and/or avoiding testosterone therapy in those with severe OSA [123, 124]. Surprisingly, other national guidelines from the United Kingdom make no mention of OSA at all [125]. The issue of testosterone effects on OSA should not be ignored because two randomized controlled trials show that testosterone therapy can acutely (within 2–3 weeks) induce sleep-disordered breathing [126, 127]. Whether these adverse findings would have occurred with longer-term near-physiological testosterone replacement is uncertain because one of the studies utilized testosterone doses that resulted in sustained supraphysiological testosterone levels [127], and the other likely induced intermittent supraphysiological peaks and assessed for OSA during these peaks [126].

Two other randomized, placebo controlled, parallel group studies have partly addressed this uncertainty [128, 129]. The first study administered a testosterone patch or a matching dose-titrated placebo patch for 3 years to 108 healthy men over the age of 65 years [128]. The initial dose was 6 mg/day, which was titrated every 3 months to maintain blood testosterone levels below 34.7 nmol/litre. No significant difference in sleep disordered breathing was detected between groups after 6, 12, 24 or 36 months of therapy. However, the method of detection was relatively insensitive and may have missed the development of mild or even moderate OSA. The second study remains the only study to purposefully administer testosterone to men with known moderate-severe OSA [129]. Sixty-seven middle-aged obese men with OSA were treated with 3 doses of testosterone undecanoate 1000 mg every 6 weeks, or matching placebo, and received recommendations for a hypocaloric diet which caused weight loss that was comparable between the two groups. Testosterone treatment significantly increased sleep-disordered breathing by a moderate amount (10 events/hour) at week 7 (one week after the second injection of testosterone undecanoate), but not at week 18. Both studies allow for the possibility that the worsening in OSA could dissipate with longer term therapy. Another possibility is that OSA is only induced acutely, potentially due to transient effects on ventilatory drive [130–132].

Despite these adverse effects on breathing during sleep, 18 weeks of testosterone therapy in men with OSA increased muscle mass, reduced liver fat, improved insulin sensitivity and heightened sexual desire compared with placebo therapy [87, 133]. Studies advancing our knowledge regarding the relative risks and benefits of testosterone therapy in older men have recently become available [134], but further research is needed. More studies examining the risks and benefits of testosterone therapy in men with OSA over the longer term are required. Until such data are available, expert opinion will appropriately continue to caution against the use testosterone therapy in men with untreated severe OSA [123].

## Summary and conclusions

Sleep is highly organized, serves important biological functions, and influences health and longevity through endocrine and metabolic regulated systems. Accumulated sleep debt is widespread in modern society and when accumulated throughout life likely impacts the ageing process, and the development of age-related diseases.

Sleep loss and lower sleep duration are associated with lower morning, afternoon and 24-h testosterone, whereas they are associated with higher late afternoon and early evening, but not morning or 24-h cortisol. These reciprocal changes in testosterone and cortisol with sleep loss imbalances catabolic-anabolic signaling and is an important, but not exclusive, mechanism by which sleep loss induces insulin resistance. By fixing testosterone-cortisol balance to prevent the induction of insulin resistance by sleep restriction, we provided the first proof-of-concept that the metabolic harm that occurs with sleep loss can potentially be mitigated by therapeutic approaches that do not require sleeping more. This approach is likely to be relevant also to older men since the changes in testosterone-cortisol balance that occur in young men have recently been shown to also occur in older men.

Epidemiological studies when considered in unison show that OSA is associated with lower testosterone levels, independently of confounders such as age and obesity. Circumstantial evidence would favor the possibility that more severe OSA, due to greater hypoxemia, lowers testosterone although the opposite is plausible. It would be premature to conclude that CPAP therapy has no effect on testosterone in men with OSA since the available studies are underpowered, and the higher quality studies suggest otherwise. In contrast, the larger number of studies available, particularly higher quality studies, has allowed the conclusion to be made that CPAP decreases cortisol. High dose testosterone therapy induces OSA, but more physiological dosing may not; this effect may be transient or dissipate with longer term therapy.

Important limitations are that only one interventional study has examined the effect of restricting sleep on testosterone and cortisol in a cohort of older men; few studies have been designed to examine changes in relevant testosterone and cortisol pulse characteristics with age and/or in response to sleep manipulation; and longitudinal epidemiological studies examining the age-related changes in sleep architecture, the impact of changes in sleep on testosterone and cortisol, are rudimentary.

Nevertheless, the available data are highly suggestive that restricted sleep, circadian misalignment and disrupted sleep from OSA are relevant to age and age-related diseases through alterations in testosterone and cortisol signaling. Therefore, society should prioritize and value sufficient and appropriately timed sleep. Future research to understand the molecular underpinnings of these findings (for example in circadian clocks [135]) is needed to develop countermeasures to reduce the impact of insufficient sleep, disrupted sleep, or circadian misalignment on cardiometabolic health. This is because insufficient sleep and night shiftwork may sometimes be unavoidable. Such investigations are currently being planned.

## Abbreviations

CPAP: Continuous positive airway pressure
GnRH: Gonadotropin-releasing hormone
LH: Luteinizing hormone
NREM: Non-rapid eye movement
OSA: Obstructive sleep apnea
REM: Rapid eye movement
SD: Standard deviation
SWS: Slow wave sleep
T2DM: Type 2 diabetes mellitus
